# Randomised controlled trial testing effectiveness of feedback about lung age or exhaled CO combined with very brief advice for smoking cessation compared to very brief advice alone in North Macedonia: findings from the Breathe Well group

**DOI:** 10.1186/s12889-023-16644-1

**Published:** 2023-09-29

**Authors:** Dragan Gjorgjievski, Katarina Stavrikj, Rachel Jordan, Peymane Adab, Gjorgji Stanoevski, Aleksandra Stamenova, Emilija Krstevska, Sara Simonovska, Fillip Trpcheski, Rachel Adams, Christina Easter, Kiran Rai, Kar Keung Cheng, Chunhua Chi, Brendan G. Cooper, Jaime Correia-de-Sousa, Andrew P. Dickens, Alexandra Enocson, Nicola Gale, Kate Jolly, Sue Jowett, Mariam Maglakelidze, Tamaz Maghlakelidze, Sonia Martins, Alice Sitch, Rafael Stelmach, Alice Turner, Siân Williams, Amanda Farley

**Affiliations:** 1 Centre for Family Medicine, Faculty of Medicine, Ss. Cyril and Methodius University in Skopje, Skopje, Republic of North Macedonia; 2 Institute of Applied Health Research, University of Birmingham, Edgbaston, Birmingham, B15 2TT UK; 3 Health Services Management Centre, School of Social Policy, University of Birmingham, Birmingham, UK; 4 Department of General Practice, Peking University First Hospital, Beijing, China; 5 Lung Function & Sleep, Queen Elizabeth Hospital, Birmingham, UK; 6 Life and Health Sciences Research Institute (ICVS), School of Medicine, University of Minho, Braga, Portugal; 7 Observational and Pragmatic research Institute, Midview City, Singapore; 8 Petre Shotadze Tbilisi Medical Academy, 51/2 Ketevan Dedofali Ave, Tbilisi, 0144 Georgia; 9 Ivane Javakhishvili Tbilisi State University, 1 Ilia Chavchavadze Avenue, Tbilisi, 0179 Georgia; 10 Family Medicine, ABC Medical School, Sao Paolo, Brazil; 11 Faculty of Medicine, University of São Paulo, São Paulo, Brazil; 12 International Primary Care Respiratory Group, 19 Armour Mews, Larbert, FK5 4FF Scotland

**Keywords:** Smoking cessation, RCT, Lung age, Carbon monoxide, Very brief advice, LMIC

## Abstract

**Introduction:**

In 2019, smoking prevalence in North Macedonia was one of the world’s highest at around 46% in adults. However, access to smoking cessation treatment is limited and no co-ordinated smoking cessation programmes are provided in primary care.

**Methods:**

We conducted a three parallel-armed randomised controlled trial (*n* = 1368) to investigate effectiveness and cost-effectiveness of lung age (LA) or exhaled carbon monoxide (CO) feedback combined with very brief advice (VBA) to prompt smoking cessation compared with VBA alone, delivered by GPs in primary care in North Macedonia. All participants who decided to attempt to quit smoking were advised about accessing smoking cessation medications and were also offered behavioural support as part of the “ACT” component of VBA. Participants were aged ≥ 35 years, smoked ≥ 10 cigarettes per day, were recruited from 31 GP practices regardless of motivation to quit and were randomised (1:1:1) using a sequence generated before the start of recruitment. The primary outcome was biochemically validated 7-day point prevalence abstinence at 4 weeks (wks). Participants and GPs were not blinded to allocation after randomisation, however outcome assessors were blind to treatment allocation.

**Results:**

There was no evidence of a difference in biochemically confirmed quitting between intervention and control at 4wks (VBA + LA RR 0.90 (97.5%CI: 0.35, 2.27); VBA + CO RR 1.04 (97.5%CI: 0.44, 2.44)), however the absolute number of quitters was small (VBA + LA 1.6%, VBA + CO 1.8%, VBA 1.8%). A similar lack of effect was observed at 12 and 26wks, apart from in the VBA + LA arm where the point estimate was significant but the confidence intervals were very wide. In both treatment arms, a larger proportion reported a reduction in cigarettes smoked per day at 4wks (VBA + LA 1.30 (1.10, 1.54); VBA + CO 1.23 (1.03, 1.49)) compared with VBA. The point estimates indicated a similar direction of effect at 12wks and 26wks, but differences were not statistically significant. Quantitative process measures indicated high fidelity to the intervention delivery protocols, but low uptake of behavioural and pharmacological support. VBA was the dominant intervention in the health economic analyses.

**Conclusion:**

Overall, there was no evidence that adding LA or CO to VBA increased quit rates. However, a small effect cannot be ruled out as the proportion quitting was low and therefore estimates were imprecise. There was some evidence that participants in the intervention arms were more likely to reduce the amount smoked, at least in the short term. More research is needed to find effective ways to support quitting in settings like North Macedonia where a strong smoking culture persists.

**Trial registration:**

The trial was registered at http://www.isrctn.com (ISRCTN54228638) on the 07/09/2018.

**Supplementary Information:**

The online version contains supplementary material available at 10.1186/s12889-023-16644-1.

## Background

In 2019, population smoking prevalence in North Macedonia was one of the highest in the world at 46% (aged 15–64) [1, 2], with 247 deaths per 100,000 attributed to smoking [1]. In some high income countries, smokers are advised to stop smoking and are supported to quit within primary care with the most effective interventions combining pharmacotherapy and behavioral support [3], interventions also known to be highly cost-effective [4, 5]. However, in North Macedonia there are few organized smoking cessation programmes available, and none in primary care. Access to pharmacotherapy is also limited due to high out of pocket costs and there is no national quit line available. Alternative methods that could increase quitting need to be tested in this population with high smoking prevalence [6].

Brief physician advice is known to be effective in prompting a quit attempt, and also leads to a small but clinically significant increase in the chance of successful quitting even without the use of pharmacotherapy [7, 8]. Health concerns can also be a motivator to consider quitting [9, 10], and presenting smokers with information about their exposure and the harmful effects of smoking may encourage quitting [11]. Lung age (LA) [12], a biomarker of premature lung ageing, and exhaled carbon monoxide (CO) levels can be non-invasively measured and immediately communicated within primary care [13, 14], and have the potential to increase the number of people attempting to quit and being successful in low and middle income (LMIC) settings.

In 2017, the International Primary Care Respiratory Group (IPRCG) led a Global Bridges funded “teach the teachers” programme in North Macedonia which trained GPs to offer very brief advice (VBA) to tobacco dependent patients. This included training to deliver behavioural support to smokers who are willing to attempt to quit smoking [15]. We evaluated the effectiveness and cost-effectiveness of combining LA or CO feedback with VBA and behavioural support for smoking cessation compared to with VBA and behavioural support alone delivered by GPs in primary care in North Macedonia who had taken part in the train the trainers programme.

## Methods

### Trial design

A multicentre three parallel-armed randomised controlled trial (RCT) with process evaluation and cost-effectiveness analysis was conducted in primary care in North Macedonia from November 2018 to May 2020. The full protocol is reported elsewhere [16].

Ethical permission was received from the ethical review board of Saints Cyril and Methodius University, North Macedonia (UKUM034/95) and institutional ethics committee at University of Birmingham (UoB), UK (ERN_18-12408). The trial was registered at http://www.isrctn.com (ISRCTN54228638) on the 07/09/2018. All methods were carried out in accordance with relevant guidelines and regulations.

### Study participants and recruitment

Thirty-eight primary care practices that had participated in the International Primary Care Respiratory Group (IPCRG)/Global Bridges “Teach the teacher” programme were trained as research sites, and 31 GP practices from both urban and rural locations in North Macedonia recruited at least 1 participant. Smokers attending primary care for any reason were given a patient information leaflet and invited to enrol if they smoked ≥ 10 cigarettes per day (cpd) and were aged ≥ 35 years old. As the aim of the interventions were to prompt a successful quit attempt, it was not a requirement for smokers to be motivated to quit before enrolling in the trial. Eligible participants who took part in the trial provided written consent [16].

### Interventions

Participants were randomised to one of three conditions. These conditions were delivered at the baseline visit which took place at an appointment, or at a re-arranged visit:

- ***Comparator - Very brief advice only (VBA)*** – Participants received very brief advice as described by the National Centre for Smoking Cessation Training (NCSCT) which had been adapted to the North Macedonia context. The adaption was developed as part of the “teach the teacher” programme in collaboration with the NCSCT [15], and in line with the IPCRG position statement on treatment of tobacco dependence [17]. The adapted version involved delivery of the three As: (1) Asking the participant if they smoked (ascertained during screening); (2) Advising about harms of smoking, benefits of quitting and the best way to stop; and Acting, where GPs asked all participants if they would like to take up the offer of support to quit smoking. The “Act” was dependent on their response. Participants responding no, or not yet, were advised that the offer of support remained available to be taken up at another time. Those responding yes were encouraged to set a quit date within a week and were offered behavioural support from the GP at 1, 2, 4 weeks and once between 8–12 weeks post-quit. The behavioural support visit protocol was based on the UK standard treatment program for smoking cessation [18]. As pharmacotherapy was not available on prescription at the time of the study, participants were advised where to purchase nicotine replacement therapies over the counter. GPs were trained to deliver the VBA and behavioural support as part of the Teach the Teacher programme and underwent a second refresher training from the research team before taking part as a research site in the trial. Regardless of quit intention, all participants were given a smoking information leaflet (supplementary file 1).
- ***Intervention - Very brief advice with feedback about lung age (VBA + LA)*** – Lung age was calculated conservatively based on the lowest of three blows into a hand-held spirometer (Vitalograph COPD-6) performed without the use of bronchodilators [12]. The reading and its implications were explained as a motivator to stop smoking as part of “advice” within VBA (supplementary file 2).
- ***Intervention - Very brief advice with feedback about exhaled CO levels (VBA + CO)*** – CO was measured with a piCO™ Smokerlyzer® (Bedfont Scientific Ltd) once. The reading in parts per million (ppm) and its implications were explained as a motivator to stop smoking as part of “advice” within VBA. Participants who attempted to quit also had their exhaled CO measurement repeated and fed back to them during their behavioural support sessions (supplementary file 3).

### Outcome measures

Participants were followed up at 4, 12 and 26wks after baseline where they completed a questionnaire to collect outcome, process and cost data. Self-reported quitting was validated using a semi-quantitative salivary nicotine test (NicAlert**™**Craig Medical Distribution Inc., CA, USA) [19], and in an exploratory analysis was validated using exhaled CO in a subset (Supplemental file 4). Electronic data were recorded in a REDCap database hosted by UoB [20, 21].

The primary outcome was the proportion of smokers who quit at 4wks (7-day point prevalence abstinence), biochemically validated with salivary cotinine (1) < 10ng/ml, or (2) < 100ng/ml for those who reported exposure to second hand cigarette smoke in the home on a daily basis, or (3) ≥ 10ng/ml in those who reported using Nicotine Replacement Therapy (NRT)/e-cigarettes at any time point during the study, irrespective of exposure to second hand cigarette smoke). Secondary outcomes were biochemically validated (as above) 7-day point prevalence abstinence at 12wks and 26wks, proportion who reported quitting smoking (self-report 7 day point prevalence abstinence), proportion who attempted to quit smoking, proportion who reduced the number of cigarettes smoked per day and motivation to quit smoking as measured by the motivation to stop smoking scale (MTSS) [22] at 4, 12 and 26wks.

### Sample size

We initially expected to find a difference of 10% in quitting at 4wks between intervention and control arms (12% VBA vs 22% VBA + LA vs 22% VBA + CO) [23]. However, this was revised in consultation with the Trial Steering Committee due to low numbers of participants attempting to quit. We finally aimed to recruit at least 1182 participants, 394 participants per arm, to detect a difference of 5% in quitting between the intervention and control arms at 4 weeks (3% VBA vs 8% VBA + LA vs 8% VBA + CO) with 80% power and a significance level of 2.5% (due to comparison of each intervention arm to the control group).

### Randomisation and blinding

The randomisation sequence was created prior to the commencement of participant recruitment and embedded within the REDCap database. The next allocation was concealed to the recruiter (the GP), and only revealed after a new participant record was created and the baseline questionnaire completed. Participants and GPs were not blinded to allocation after randomisation, however outcome assessors were blind to treatment allocation. Participants were randomised 1:1:1, stratified by GP practice.

### Statistical methods

Data were analysed using Stata (version 16, Texas, USA) [24]. Baseline measures were reported as frequency and percentages for binary measures or mean and standard deviation or median and interquartile range (25% percentile and 75^th^ percentile) for continuous measures where appropriate. Primary and secondary outcomes were analysed using Poisson regression models with robust standard errors adjusting for primary care practice as a random effect. Model estimates were reported as relative risks (RR) with confidence intervals (97.5% for the primary analysis and 95% for all other analyses). In accordance with the Russell Standard [25], an intention to treat analysis was conducted for smoking cessation outcomes, treating those lost to follow-up as smokers, and untraceable participants were removed from the analysis.

Planned sub-group and sensitivity analyses [16] were not undertaken due to the small numbers of participants who quit. Exploratory analyses conducted at 4, 12 and 26wks compared alternative definitions of the primary outcome in a subset of participants. Alternative definitions used different criteria for cotinine testing and used exhaled CO in place of cotinine for biochemical validation (supplemental file 4). This was conducted in order to explore the impact on the primary outcome of accounting for the use of nicotine replacement products or exposure to secondhand smoke.

### Process evaluation

A process evaluation to describe fidelity in intervention delivery and also uptake was conducted using data captured within case report forms (CRF). This included describing the proportion with LA and CO measurements recorded, proportion setting a quit date, length of time between baseline and quit date, number of behavioural support sessions attended, and use of pharmacotherapy at any time point during the study. We also captured audio recordings of a sample of participant baseline visits in order to assess fidelity in intervention delivery.

### Cost effectiveness analysis

An incremental cost-effectiveness analysis was used to calculate cost per additional quitter at 4wks for both interventions, and a cost-utility analysis conducted to calculate cost per quality-adjusted life year (QALY) gained over 26wks, using data from the EQ-5D-5L questionnaire [26].

## Results

### Baseline characteristics

**Fig. 1:**
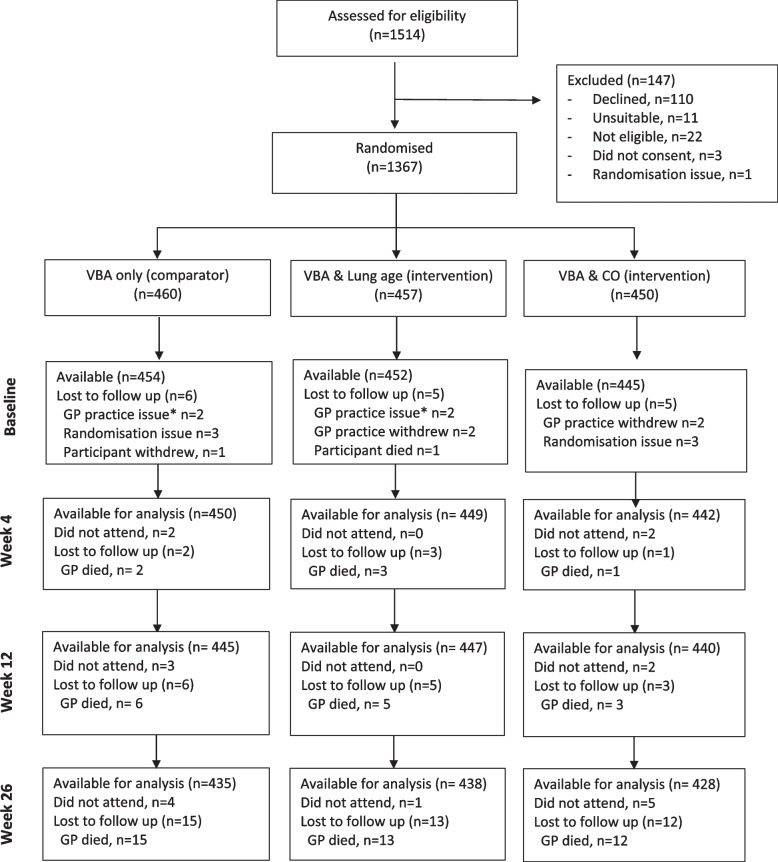
CONSORT diagram for the flow of participants. According to the Russel standard [25], participants who did not attend but were contactable remained in the study and were counted as smokers. Participants that were not contactable (i.e. moved with no forwarding contact details, unobtainable phone number, had died) were excluded from the denominator and not available for analysis. *Practice excluded due to protocol deviation

We assessed 1514 patients for eligibility and randomised 1367 into the study (VBA + LA (*n* = 457), VBA + CO (*n* = 450) or VBA (*n* = 460)). The flow of participants through the study is summarised in Fig. 1. Characteristics of included participants were well balanced between arms (Table 1). Overall, mean age was 51 years and 47.6% were male. Eligible patients who did not want to participate were slightly older (mean age 55 (SD 11) and more likely to be male (59.6%).

**Table 1:** Baseline characteristics of participants by treatment group

**Characteristics**	**VBA only (*n* = 454)**	**VBA+ lung age (*n* = 452)**	**VBA + exhaled CO (*n* = 445)**
Male sex, n (%)	212 (46.7)	220 (48.7)	211 (47.4)
Age (years), mean (SD)	50.74 (10.23)	50.92 (10.33)	51.68 (10.42)
Macedonian ethnicity, n (%)	384 (84.6)	366 (81.0)	376 (84.5)
Employment, n (%)			
Employed	303 (66.7)	309 (68.4)	296 (66.5)
Unemployed	48 (10.6)	52 (11.5)	50 (11.2)
Retired	64 (14.1)	68 (15.0)	75(16.9)
Unable to work	35 (7.7)	22 (4.9)	21 (4.7)
Cigarettes smoked per day, mean (SD)	20 (10)	19 (10)	19 (11)
Roll cigarettes per day, n (%)			
0	279 (61.5)	268 (59.3)	272 (61.1)
1–10	9 (2.0)	7 (1.6)	5 (1.1)
11–20	35 (7.7)	38 (8.4)	35 (7.9)
≥ 21	28 (6.2)	36 (8.0)	24 (5.4)
Serious quit attempts, n (%)			
Yes	236 (52.0)	235 (52.0)	251 (56.4)
No. serious attempts, n (%) of people making serious attempt			
0	1 (0.4)	0 (0)	3 (1.2)
1–10	229 (97.0)	231 (98.3)	240 (95.6)
> 11	3 (2.5)	2 (0)	3 (1.1)
Motivated to quit, n (%)	29 (6.4)	27 (6.0)	23 (5.2)
Fagerström test for nicotine dependence (FTND) score, mean (SD)	5.09 (2.3)	5.05 (2.3)	4.88 (2.3)
Co-morbidities, n (%)	268 (59)	298 (66)	262 (59)
Income (MKD), n (%)			
< 10,000	41 (9.0)	40 (8.9)	29 (6.5)
10,000–19,999	122 (26.9)	129 (28.5)	132 (29.7)
20,000–29,999	101 (22.3)	84 (18.6)	108 (24.3)
> 30,000	92 (20.3)	103 (22.8)	85 (19.1)
Prefer not to say	96 (21.2)	92 (20.3)	89 (20.0)
Living in a City^a^, n (%)	358 (78.9)	342 (75.7)	333 (74.8)
Education level, n (%)			
No formal qualifications	4 (0.9)	4 (0.9)	5 (1.1)
Primary	68 (15.0)	79 (17.5)	74 (16.6)
Secondary	244 (53.7)	238 (52.7)	245 (55.1)
Tertiary	71 (15.6)	77 (17.0)	59 (13.3)
Visho level^b^	45 (9.9)	41 (9.1)	46 (10.3)
Postgraduate degree	21 (4.6)	11 (2.4)	13 (2.9)
Other	1 (0.2)	2 (0.4)	2 (0)

^a^Reference category is living in a village

^b^Bachelor degree or ISCED 5 code (tertiary education)

### Primary outcome

In total, 23 of 1351 (1.7%) participants were biochemically validated as abstinent from smoking at 4wks (VBA + LA *n* = 7/449, 1.6%, VBA + CO *n* = 8/442, 1.8%, VBA *n* = 8/450, 1.8%). Compared to VBA, the point estimate showed a lower validated quit rate in the VBA + LA arm (RR 0.90 (97.5% CI: 0.35, 2.27)) and a slightly higher quit rate for VBA + CO (RR 1.04 (97.5%CI: 0.44, 2.44)) (Table 2). However, for both interventions, CIs were wide and therefore estimates are imprecise.

**Table 2:** Proportion of participants who quit smoking (biochemically confirmed and self-reported) and proportion who reduced the number of cigarettes smoked per day

	**VBA only**		**VBA + lung age**		**VBA + exhaled CO**		**VBA + lung age**		**VBA + exhaled CO**	
	**Total**	**n (%)**	**Total**	**n (%)**	**Total**	**n (%)**	**RR (95% CI)**	***p*-value**	**RR (95% CI)**	***p*-value**
**Quit smoking (biochemically confirmed)**										
At 4wks^a^	450	8 (1.8)	449	7 (1.6)	442	8 (1.8)	0.90 (0.35–2.27)^b^	0.792	1.04 (0.44–2.44)^b^	0.920
At 12wks	445	8 (1.8)	447	6 (1.3)	440	10 (2.3)	0.78 (0.32–1.89)	0.578	1.29 (0.65–2.57)	0.463
At 26wks	435	1 (0.2)	438	7 (1.6)	428	3 (0.7)	6.98 (1.09–44.54)	0.040	3.11 (0.28–35.10)	0.358
**Quit smoking (self-reported)**										
At 4wks	450	8 (1.8)	449	9 (2.0)	442	15 (3.4)	1.15 (0.51–2.59)	0.731	1.95 (0.92–4.10)	0.080
At 12wks	445	11 (2.4)	447	11 (2.4)	439	18 (4.1)	1.02 (0.45–2.30)	0.970	1.69 (0.73–3.91)	0.218
At 26wks	435	11 (2.5)	437	17 (3.9)	428	15 (3.5)	1.58 (0.74–3.38)	0.243	1.41 (0.65–3.08)	0.391
**Reduction in cpd**										
At 4wks	389	110 (28.3)	386	141 (36.5)	383	132 (34.5)	1.30 (1.10–1.54)	0.002	1.23 (1.03–1.49)	0.026
At 12wks	385	143 (37.1)	380	160 (42.1)	376	152 (40.4)	1.15 (1.00–1.32)	0.048	1.11 (0.93–1.32)	0.240
At 26wks	372	172 (46.2)	365	173 (47.4)	366	182 (49.7)	1.03 (0.94–1.14)	0.507	1.09 (0.95–1.26)	0.225

All analyses adjusted for primary care research site as a random effect

*RR* Relative Risk, compared with VBA only, *cpd* Cigarettes smoked per day

^a^Primary outcome: Proportion of smokers who are quit at 4 weeks (7-day point prevalence self-reported abstinence), confirmed with salivary cotinine level of: (1) < 10ng/ml, or (2) < 100ng/ml for those who report SHS exposure in the home on a daily basis, or (3) ≥ 10ng/ml in those who report using NRT/e-cigarettes at any time point during the study, irrespective of second-hand smoke exposure

^b^Confidence interval for primary outcome is 97.5%

### Secondary outcomes

#### Biochemically validated and self-reported quitting

The total number of validated quitters at 12wks and 26wks was low (12wks: 24/1332 = 1.8%; 26wks: 11/1301 = 0.8%). Compared to VBA, point estimates for validated quit rates were lower in the VBA + LA arm at 12wks (RR 0.78 (95% CI:0.32, 1.89) but higher at 26wks (RR 6.98 (95% CI:1.09, 44.54). In the VBA + CO arm, rates were higher at both 12wks (RR 1.29 (95% CI:0.65, 2.57)) and 26wks (RR 3.11 (95% CI:0.28, 35.10)). However, the confidence interval for all estimates were also wide and included no effect apart from LA at 26 weeks where the point estimate was significant, but confidence intervals were very wide (Table 2).

For self-reported quitting, point estimates for quit rates were higher in the VBA + LA and VBA + CO arm in comparison to VBA at 4wks (RR 1.15 95% CI:0.51, 2.59; RR 1.95 95% CI:0.92, 4.10;), 12wks (RR 1.02 95% CI: 0.45, 2.30; RR 1.69 95% CI: 0.73, 3.91) and at 26wks (RR 1.58 (95% CI: 0.74, 3.38; RR 1.41 (95% CI: 0.65, 3.08)). However, these estimates also did not reach statistical significance (Table 2).

#### Reduction in cigarettes smoked per day

There was a relative increase in participants reporting reduction in the number of cigarettes smoked per day in VBA + LA and VBA + CO arms compared to VBA at all follow up points. At 4wks this was statistically significant in both the VBA + LA arm (RR 1.30 (95% CI: 1.10, 1.54)) and the VBA + CO arm (RR 1.23 (95% CI: 1.03, 1.49)). At 12wks (RR 1.15 (95% CI: 1.00, 1.32), (RR 1.11 (95% CI: 0.93, 1.32)) and 26wks (RR 1.03 (95%CI: 0.94, 1.14)), (RR 1.09 (95%CI: 0.95, 1.26)) CIs included no effect (Table 2).

#### Motivation and attempts to quit smoking

Motivation and attempts to quit smoking broadly followed the same pattern as validated quit rates. In the VBA + LA arm, fewer were motivated and had attempted to quit compared to the VBA arm at earlier timepoints but more at 26wks. In the VBA + CO arm, a higher proportion were motivated to quit and had attempted quitting at all three timepoints compared with the VBA arm, but only attempts to quit at 12wks were significantly higher (RR 1.62 (95% CI: 1.10, 2.39)) (Table 3).

**Table 3:** Motivation and attempts to quit smoking during the follow-up time points

	**VBA only**		**VBA + lung age**		**VBA + exhaled CO**		**VBA + lung age**		**VBA + exhaled CO**	
	**Total**	**n (%)**	**Total**	**n (%)**	**Total**	**n (%)**	**RR (95% CI)**	***p*-value**	**RR (95% CI)**	***p*-value**
**Motivation to quit**										
At 4wks	446	25 (5.6)	438	24 (5.5)	435	33 (7.6)	0.98 (0.62–1.54)	0.922	1.35 (0.88–2.05)	0.167
At 12wks	442	26 (5.9)	444	23 (5.2)	436	30 (6.9)	0.89 (0.56–1.42)	0.622	1.18 (0.75–1.85)	0.483
At 26wks	430	28 (6.5)	435	32 (7.4)	425	31 (7.3)	1.14 (0.74–1.73)	0.557	1.12 (0.75–1.66)	0.581
**Attempting to quit**										
At 4wks	448	38 (8.5)	446	35 (7.9)	439	48 (10.9)	0.94 (0.66–1.33)	0.723	1.30 (0.97–1.76)	0.080
At 12wks	443	31 (7.0)	443	42 (9.5)	435	48 (11.0)	1.39 (0.94–2.04)	0.098	1.62 (1.10–2.39)	**0.016**
At 26wks	430	49 (11.4)	435	64 (14.7)	424	59 (13.9)	1.29 (0.91–1.84)	0.155	1.24 (0.87–1.76)	0.246

All analyses adjusted for primary care research site as a random effect

*RR* Relative Risk compared with VBA only adjusting for baseline data

Proportion ranking as I REALLY want to stop smoking and intend to in the next 3 months (MTSS=6), I REALLY want to stop smoking and intend to in the next month (MTSS=7) or I have stopped smoking

### Process measures

Of 1351 participants with baseline measurements, 65 (4.8%) set a quit date. The median length of time between randomisation and quit date was 6 days (interquartile range = 2–8). Twenty-two (33.8%) of those setting a quit date used NRT or e-cigarettes. The mean number of behavioural support visits was 2.43 (SD = 1.73) out of a total possible of 5 visits. The majority of participants were recorded within the CRF as receiving VBA across all trial arms (98.7–99.8%), and the CO (99%) and LA components (98%) in the intervention arms.

Thirty-three baseline visits were recorded capturing intervention delivery. Recordings also indicated that the LA and CO components were delivered with high fidelity, whereas fidelity to the VBA protocol was higher in the VBA-only arm (supplementary file 5).

### Health economic evaluation

Overall, the costs of the VBA intervention were lower at 114.67 MKD per patient compared with 119.80 and 136.29 for the VBA + LA and VBA + CO arms respectively. Furthermore, the VBA arm had slightly more quitters at the 4wk primary endpoint and slightly higher total QALYs over 26wks (0.4525 (SD 0.0514)), resulting in VBA being the dominant intervention (cheaper and more effective than other approaches). Few patients reported expenditure on products to stop smoking, but this expenditure was high for the 31 reporting it—3000–4000 MKD (Table 4).

**Table 4:** Descriptive health outcomes and costs. Values are mean (SD) unless otherwise stated

**Health outcomes**	**VBA**	**VBA + Lung age**	**VBA + CO**
Baseline EQ-5D-5L	0.871 (0.166) *n* = 446	0.883 (0.149) *n* = 444	0.885 (0.152) *n* = 438
4-wk EQ-5D-5L	0.898 (0.146) *n* = 448	0.911 (0.142) *n* = 446	0.901 (0.142) *n* = 439
12-wk EQ-5D-5L	0.900 (0.149) *n* = 442	0.908 (0.147) *n* = 444	0.913 (0.134) *n* = 435
26-wk EQ-5D-5L	0.912 (0.134) *n* = 426	0.906 (0.145) *n* = 432	0.909 (0.138) *n* = 425
**Total QALYs over 6 months**	*n* = 418	*n* = 423	*n* = 414
Unadjusted	0.4497 (0.0661)	0.4525 (0.0666)	0.4532 (0.0630)
Incremental QALYs compared with VBA (95% CI)		0.0028 (-0.0060 to 0.0117)	0.0035 (-0.0053 to 0.0124)
Adjusted QALY (SD)^a^	0.4525 (0.0514)	0.4519 (0.0462)	0.4510 (0.0461)
Incremental QALYs compared with VBA (95% CI)^a^		-0.0005 (-0.0065 to 0.0055)	-0.0015 (-0.0075 to 0.0046)
**Costs**	VBA only	VBA + Lung age	VBA + CO
Intervention cost per patient(MKD)	6.34	58.35	30.01
Spent money on products to stop smoking (*n* =)	*n* = 418 12	*n* = 423 8	*n* = 414 11
Mean amount spent on products to stop smoking over 6 months (MKD):			
- All patients	108.33 (848.97)	61.45 (552.68)	106.28 (798.61)
- Patients who reported spending money	3773 (3498)	3250 (2563)	4000 (3074)
**Total 6 month cost (intervention plus patient expenditure on products) (MKD)**	**114.67**	**119.80**	**136.29**

^a^Adjusted for baseline EQ-5D-5L score

## Discussion

This study tested the effectiveness of simple interventions to prompt smoking cessation delivered to smokers in primary care in North Macedonia. As these were interventions advising smokers to quit, they were delivered to all smokers, regardless of motivation to quit smoking, with the aim of prompting a successful quit attempt. Point estimates indicated that biochemically validated quit rates, self-reported quit rates, motivation to quit, attempts to quit and the proportion reducing the number of cigarettes smoked per day were higher in the CO + VBA arm compared to VBA at all follow up points, whereas point estimates were more inconsistent for the LA + VBA arm. However, it was not possible to draw conclusions about effectiveness of the interventions as the absolute number of participants quitting was lower than expected and estimates were imprecise incorporating the possibility of no effect, with the exception of the proportion who had reduced the number of cigarettes smoked at 4wks and attempts to quit at 12wks in the VBA-CO arm. After taking into account the costs incurred and QALYs gained, the health economic analysis indicated that VBA alone was the dominant intervention (less costly, more effective) however interpretation of this is also limited due to negligible differences in QALYs.

### Strengths and limitations

This is the first trial testing effectiveness of interventions to prompt smoking cessation in primary care in North Macedonia [27], a middle income country with high smoking prevalence and limited tobacco control measures in place [1, 6]. Given the limited availability of affordable pharmacotherapy, we sought to test other interventions which could feasibly be delivered in primary care. These were identified with local stakeholders through a prioritisation exercise conducted before designing the trial [28] and we were able to build on an existing in-country programme training general practitioners to deliver VBA for smoking cessation with behavioural support for smokers who choose to quit (IPCRG/Global Bridges teach the teacher programme) [15]. There are no data describing demographic characteristics of smokers in North Macedonia, however characteristics of participants were similar to those of the general population, and eligible patients declining participation were not substantially different to trial participants. Primary care practices from both rural and urban areas participated in the trial and attrition bias was minimised with high follow up rates in each arm. Therefore, an important strength of the study is that generalisability of the findings to the smoking population in North Macedonia is likely to be high.

A challenge with this study was the ability to accurately predict expected quit rates in the control and intervention arms. One study testing LA + VBA in smokers regardless of motivation to quit and reporting quit rates at 4wks was found when we were designing the trial, and this had been conducted in Ireland [23]. Our initial sample size calculation, which was based on this Irish study, was revised as advised by the Trial Steering Committee due to a lower observed proportion attempting to quit in our study. An increased recruitment target was approved, with an expectation of 3% quit at 4wks in the VBA arm and increase of 5% in the intervention arms. However, these expected rates were also not met and although we exceeded our recruitment target, the trial may not have been adequately powered to detect a difference in the primary outcome. A statistically significant difference was seen in the VBA CO arm in reduction in cigarettes smoked per day and in attempts to quit, but it should be noted that even when reducing the number of cigarettes smoked per day, smokers may not experience a reduction in harm due to compensatory smoking [29].

### Comparison with literature

The available evidence on effectiveness of delivering LA or exhaled CO feedback on smoking cessation is summarised in a Cochrane review published in 2019 [11]. This reported moderate certainty evidence from five studies that feedback on CO measurement did not increase quitting at 6 months (RR 1.00 (95% CI 0.83 to 1.21); I^2^ = 0%; *n* = 2368). When designing the trial, we considered that this finding may not be replicated in North Macedonia as these studies were all conducted in high income countries (HICs) and did not repeat CO measurements during behavioural support as was the case in our study. In addition, three out of five of the studies were at high risk of bias. However, we did not find evidence to the contrary suggesting that use of CO measurements as an augmented aspect of advice in VBA and repeated as part of behavioural support in those who attempt to quit is not effective within the North Macedonia context.

The Cochrane review also included two studies testing LA. The first study was small (*n* = 50) and did not show a positive effect [30]. The second was a trial conducted in 561 smokers in primary care that used spirometry and immediate feedback of lung age using a graphical display [14]. Participants receiving the intervention were told their LA if this was older than their chronological age and participants in the control arm received the raw spirometry reading. Contrary to our findings, the study found evidence of an increase in quitting (Intervention 13.6% v control 6.4% (RR 2.12 (1.24, 3.62) at 12 months). However, this study was conducted in a HIC, and may not be transferable to the North Macedonian context.

VBA is recommended as standard care in primary care in LMIC settings by the World Health Organisation [31]. Although some GPs may deliver VBA, at the time of this study there was no national clinical tobacco guidance in North Macedonia. Evidence from a Cochrane review shows that brief advice for smoking cessation delivered by physicians in primary care is effective (OR 1.66 (95% CI 1.42–1.94)) [8], and has a small but significant long term effect on quitting in absolute terms that are deemed to be clinically important (~ 2% increase above no intervention, with absolute quit rates of ~ 4% after at least 26 weeks) [7, 8]. Many of the trials testing brief advice included in the Cochrane review were conducted decades ago in the US or UK when smoking prevalence was closer to the current prevalence in North Macedonia and implementation of tobacco control measures was similarly less well advanced. It would therefore be reasonable to expect that brief advice delivered in the North Macedonia context may have similar effectiveness. Despite this, our study found that only 1/435 (0.2%) smokers who received VBA were quit at 26wks, and the quit rate was also lower than expected at 4wks. There are few studies testing VBA delivered by healthcare providers in LMIC settings for comparison [27, 32]. However, two small studies conducted in Malaysia [33] and China [34] reported a 15% quit rate in smokers unselected by motivation or pre-existing health condition at 6 and 12 months, respectively. This suggests that absolute quit rates in North Macedonia may be particularly low, and more work is required to understand how to increase the impact of VBA from physicians in this context.

There are a number of factors that may have contributed to the low absolute quit rates seen within our study. In terms of fidelity to intervention delivery, quantitative measures taken across all trial participants indicated that this was high for the LA, CO and VBA protocols, however recordings of a sample of baseline consultations indicated that fidelity to the VBA protocol was reduced for the lung age and CO arms compared to the VBA arm alone. It is possible that delivery of the LA and CO components distracted GPs from delivering the full VBA protocol, and this may have masked differences in effectiveness between these approaches. In line with the protocol, participants setting a quit date did so within a week of the baseline visit. However, uptake of behavioural support was low with participants receiving an average of two out of a possible five sessions offered as part of the standard programme for behavioural support, and only 34% indicated that they had used some form of pharmacotherapy.

In addition to these reasons, generally speaking, smoking remains a strong part of the culture within North Macedonia. National smoking prevalence is high [35–37], including among healthcare workers [38]. It is the fifth largest producer of raw tobacco leaf in Europe, representing 13.9% of European tobacco product [36], and a fifth of our GP practices were based in tobacco producing areas. North Macedonia are a signatory country to the WHO Framework Convention on Tobacco Control, but tobacco prices remain affordable, there is not a complete ban of smoking in public places, there is no standardised packaging of cigarettes or mass media campaign, and availability of support services and pharmacotherapies is low [38].

The low numbers of quitters in our study may mean that our trial was underpowered, and small effects of brief interventions in primary care cannot be ruled out. However, the findings highlight the difficulty of influencing smokers through primary care services within North Macedonia alone. Tackling high national smoking prevalence will also be reliant on more complete adoption of the WHO FCTC population based tobacco control measures, and on making known effective pharmacotherapies more accessible and affordable. More research is needed to find effective ways to prompt and support quitting in primary care in North Macedonia.

## Conclusion

There was insufficient evidence to draw strong conclusions regarding the effectiveness of adding LA and CO to VBA for smoking cessation when delivered in primary care in North Macedonia. Overall, absolute quit rates were much lower than reported in HIC and other LMIC settings and confidence intervals were wide including the possibility of no effect. However, there was evidence of willingness to attempt quitting in some smokers. Further work is needed to identify barriers to successful quitting and to find effective and cost-effective methods to support patients to quit smoking in primary care in North Macedonia.

### Supplementary Information

**Additional file 1: Supplementary file 1.** VBA Protocol including behavioural support for smokers who choose to quit. **Supplementary file 2.** Protocol for conducting lung age test and explaining significance to the participant. **Supplementary file 3.** Protocol for conducting CO test and explaining significance of the result to the participant. **Supplementary file 4.** Exploratory analyses (validation method for quitting smoking). **Supplementary file 5.** Process evaluation - CRF measures and fidelity recordings.

## Data Availability

Data are available subject to reasonable request to the corresponding author Rachel Jordan (r.e.jordan@bham.ac.uk), and further ethical approvals.

## Abbreviations

LA: Lung age
CO: Carbon Monoxide
VBA: Very brief advice
LMIC: Low and middle income
IPCRG: International Primary Care Respiratory Group
RCT: Randomised Controlled Trial
UoB: University of Birmingham
NCSCT: National Centre for Smoking Cessation Training
CRF: Case report form
QALY: Quality-adjusted life year
RR: Relative risk
MKD: Macedonia Denar
MTSS: Motivation to Stop Scale
