# Supplementary material for: Randomised controlled trial testing effectiveness of feedback about lung age or exhaled CO combined with very brief advice for smoking cessation compared to very brief advice alone in North Macedonia: findings from the Breathe Well group
Source: BMC Public Health. 2023 Sep 29;23:1887. doi: 10.1186/s12889-023-16644-1 (PMC10541684; doi:10.1186/s12889-023-16644-1)
Supplement: Supplementary file 1 — Additional file 1: Supplementary file 1. VBA Protocol including behavioural support for smokers who choose to quit. Supplementary file 2. Protocol for conducting lung age test and explaining significance to the participant. Supplementary file 3. Protocol for conducting CO test and explaining significance of the result to the participant. Supplementary file 4. Exploratory analyses (validation method for quitting smoking). Supplementary file 5. Process evaluation - CRF measures and fidelity recordings. [file 12889_2023_16644_MOESM1_ESM.docx]

**Supplementary file 1**

**VBA Protocol including behavioural support for smokers who choose to quit.**

***In this text, we would like to inform you, the doctors who are taking part in this important project, in order to conduct the VBA Protocol with your patients in the best way and how to interpret the results as part of a smoking cessation advice.***

1. **ASK (** Establishing and recording the patient smoking status **)**

**Every patient should be asked about the smoking status every time we have an opportunity.**

**The doctors who participate in this project will ask the patients the questions below:**

**(The doctors who participate in this project have to know the smoking status of their patients. According the information from the patient file:**

1. **If the doctor doesn’t know the smoking status of the patient he should ask the patient**
   - **“Do you smoke either manufactured or roll-up cigarettes?”**
2. **ADVISE (**Clear, non-aggressive, non-judgmental advise for smoking cessation)

**In this section first of all the doctors will tell the patients the damage of smoking tobacco and the benefits of quitting smoking. In this section the doctor should bring the facts in non-judgmental way to avoid a negative attitude from the patient. In order to achieve greater success in presenting the facts, a physician needs to connect them to a certain health condition or situation that is important to the patient. In this way, the doctor will attract the patient's attention and make them think about the benefits of quitting smoking.**

**All the doctors will tell next most important benefits of quitting smoking or damage of smoking.**

- - Nobody likes a dirty mouth. After a few days without cigarettes, your smile will be brighter. Quitting smoking now will keep your mouth healthy for years to come.
  - Smoking is the leading cause of heart attacks and heart disease. But many of these heart risks can be reversed simply by quitting smoking. Quitting can lower your blood pressure and heart rate almost immediately. Your risk of a heart attack declines within 24 hours.
  - Your lungs can be very badly affected by smoking. Coughs, colds, wheezing and asthma are just the start.
  - Smoking reduces the amount of oxygen that gets to your skin. This means that if you smoke, your skin ages more quickly and looks grey and dull. The toxins in your body also cause cellulite. Quitting can help clear up blemishes and protect your skin from premature aging and wrinkling.
  - Smoking can cause male impotence, as it damages the blood vessels that supply blood to the penis. It can also damage sperm, reduce sperm count and cause testicular cancer.( If the patient is male)
  - For women, smoking can reduce fertility. (If the patient is female)

**After bringing some facts related with smoking tobacco, the doctor will,** in intervention arm only, - CO testing /Lung age - perform exhaled CO test, give results and explain significance, perform lung age measurements, give results and explain significance. (The explanation for the results of these two methods is in the supporting documents).

**And give non-aggressive, non-judgmental advice for smoking cessation:**

- Smoking is a serious health risk, but also addiction which you can beat
- Quitting smoking is not easy, but it is the single most important thing you can do to protect your health now and in the future. We are here to help you
- New findings show that the best method of smoking cessation combines drugs and medical support.
- It is important that you quit smoking now, and I can help you.

Smoker should be asked if he/she have considered/or want to quit smoking

**“Have you thought about giving up smoking?”**

The smoker can give us 3 possible answers:

1. No, I do not.
2. I have considered/ I want to quit, but not now.
3. Yes I have considered/Yes I want to quit smoking.

Knowing this answers the doctor is educated to give 3 types of reaction/ intervention in the next level ACT.

1. **ACT** (Health intervention according the previous possible answers)

**(In this section the doctor should ACT according the answer from the previous question**

**If the patient said NO, not ready then the doctor will tell**

- “I respect your decision, but if you consider that you will change your mind we are here to help you”,
- “As your doctor, I'm obliged to return to this topic because smoking is a serious health risk”. When you come to my office again I’ll ask you once again to see if you have changed your mind”.

**If the patient said YES, but not yet...unsure** then the doctor will asked for assistance

- “OK, but it will not be easier in the future. In fact, the longer you smoke, the waiver is more difficult. Whenever you decide to do it, I can offer my support also I respect your decision, but if you consider that you will change your mind we are here to help you”.

**If the patient said YES, ready to quit** than the doctors Congratulate the patients.

Try to determinate quitting date and explain the rule ,,not a single puff”. (with this rule the patient has a better chances to quit smoking and less chances for possible relapses).

Together with the patient make an individual plan for medical support and determine the first consultation for smoking cessation.

**Quit date (This section explain what doctor should say to the patients about preparations of quit date)**

Here are some steps to help patients get ready for there Quit Day:

- Pick the date and mark it on your calendar.
- Tell friends and family about your Quit Day.
- Get rid of all the cigarettes and ashtrays in your home, carand at work.
- Stock up on oral substitutes – sugarless gum, carrot sticks, hard candy, cinnamon sticks, coffee stirrers, straws, and/or toothpicks.
- Practice saying, “No thank you, I don’t smoke.”
- Set up a support system. This could be a group program or a friend or family member who has successfully quit and is willing to help you.
- Ask family and friends who still smoke not to smoke around you, and not to leave cigarettes out where you can see them.
- Think about your past attempts to quit. Try to figure out what worked and what didn’t.
- From the ,,Quit date” on you must commit to the rule ,,not a single puff”

Successful quitting is a matter of planning and commitment, not luck. Decide now on your own plan.

Over time, smoking becomes a strong habit. Daily events, like waking up in the morning, finishing a meal, drinking coffee, or taking a break at work, often trigger your urge to smoke. Breaking the link between the trigger and smoking will help you stop.

On your Quit Day go down this list:

- Do not smoke. This means not at all – ,,not a single puff”!
- Stay busy – try walking, short bursts of exercise, or other activities and hobbies.
- Drink lots of water and juices.
- Start using nicotine replacement if that’s your choice.
- Attend a stop-smoking class or follow your self-help plan.
- Avoid situations where the urge to smoke is strong.
- Avoid people who are smoking.
- Drink less alcohol or avoid it completely.
- Think about how you can change your routine. Use a different route to go to work. Drink tea instead of coffee. Eat breakfast in a different place or eat different foods.

Be prepared to feel the urge to smoke. It will pass whether you smoke or not. Use the 4 D’s to help fight the urge:

- **Delay** for 10 minutes. Repeat if needed.
- **Deep breathe.** Close your eyes, slowly breathe in through your nose and out through your mouth. Picture your lungs filling with fresh, clean air.
- **Drink water** slowly, sip by sip.
- **Do something else.** Some activities trigger cravings. Get up and move around.

Often this simple trick will allow you to move beyond the strong urge to smoke.

**First Consultation – in a week**

- Check on progress – quitting plan
- Reinforce the ,,not a single puff”rule if necessary
- Ask about withdrawal symptoms, specifically urges to smoke, and how patient has dealt with them
- Review medication usage and supply (if use any)
- To discuss and assist in dealing with barriers, if any, during the past week ( fear of failure, stress coping, weight gain, social pressure), advice and possible solutions
- Possible option for support through telephone consultation
- Arrange the next consultation – in 2 weeks

**2^nd^ Consultation – in 2 weeks**

- Check on progress – quitting plan ( the patient is disciplinary in quitting plan )
- Reinforce the ,,not a single puff”rule if necessary
- Ask about withdrawal symptoms, specifically urges to smoke, and how patient has dealt with them
- Review medication usage and supply (if use any)
- To discuss and assist in dealing with barriers, if any, during the past 2weeks,(fear of failure, stress coping, weight gain, social pressure), advice and possible solutions
- Possible option for support through telephone consultation
- Arrange the next consultation – in 4 weeks

**3^rd^ Consultation – in 4 weeks**

- Check on progress – quitting plan (the patient is disciplinary in quitting plan)
- Reinforce the ,,not a single puff”rule if necessary
- Ask about withdrawal symptoms, specifically urges to smoke, and how patient has dealt with them
- Review medication usage and supply (if use any)
- To discuss and assist in dealing with barriers, if any, during the 4 weeks,( fear of failure, stress coping, weight gain, social pressure)
- ( се препорачува оваа консултација да се одвива во амбуланта)Arrange the next consultation – 8-12 weeks

**4^th^ Consultation after 8-12 weeks**

- Check on progress – quitting plan, motivation, satisfaction **“status of a non-smoker” (**if the patient was disciplinary in quitting plan)
- Congratulate them on their success ,praise them
- Reinforce the ,,not a single puff”rule if necessary
- Review medication usage (if use any), reduction or exclusion, effects and side effects, the need for continuing treatment
- Discuss the symptoms that have occurred in the past period and offer appropriate solutions
- To discuss and assist in dealing with barriers.
- Allow the patient to describe the positive effects of smoking cessation give him time
- Joint review of the individual plan of the patient.
- Support through telephone consultation

At the end of the process we will have 2 possible results:

a. Ex-smoker - a patient that still needs our medical support. Congratulate and encourage this patient every time you have opportunity and motivate him/her with the rule ,,not a single puff”.

b. Smoker- a patient that didn’t succeed. Every time the doctor has opportunity should use VBA for smoking cessation.

# Supplementary file 2

# Protocol for conducting lung age test and explaining significance to the participant

### Procedure

1. Both patients and practitioners should use a non-alcohol sanitiser gel on their hands before the test
2. Explain background information to patient
3. Measure height
4. Turn the machine on
5. Enter height, age and gender into the microspirometer
6. Briefly explain what you would like your client to do
7. Demonstrate the right way to the patient
8. Ask the patient to attach a mouthpiece and to do the blow
9. Repeat for a total of 3 blows
10. The mouthpiece can be disposed of by the client (they can remove the mouthpiece themselves and place it in a bin)

Infection control procedures generally include:

1. Washing your hands and/or using a non-alcohol sanitising gel before and after carrying out the CO test
2. Asking the patient to place the disposable cardboard tube onto the plastic mouthpiece prior to the test
3. Asking the patient to dispose of the cardboard tube once the test has been completed
4. Wiping the CO monitor with a non-alcohol wipe between CO tests

### Suggested phrases to patient – Baseline visit

**Prior to test – background information**

*“From this graph you can see that lung function normally reduces gradually with age. Smoking can damage lungs as if they are ageing faster than normal; as an example see the line for the person with real age of 52 who has smoked and therefore has the lungs of a 75 year old. It is possible to measure the functioning of your lungs and see if they are older than would be expected for your age. Although it is not possible to repair the damage that is already done, smoking cessation slows down the rate of deterioration of the lung function back to normal, so your lungs starts to “age” normally again.*

**[Insert Figure 1 from BMJ 2008; 336:598]**

**During the test**

*“I want to measure how hard and fast you can blow out. Breathe normally, then when I tell you: take a* ***giant*** *deep breath in and blow out as* ***hard*** *and as* ***fast*** *as you can until you are* ***completely empty*** *or until I tell you to stop. Make sure you have a tight seal on the mouthpiece with your lips tightly around it. You can rest your teeth on the mouthpiece.”*

**After the test – Explain the significance of the test results**

From the 3 readings, give the LOWEST lung age reading to the patients

If the lung age is equal to or less than the individual’s chronological age, tell the participant:

*“The test result is normal, however it is important to stop smoking before the damage is done. “*

If lung age is greater than chronological age, tell the participant:

*“Your lung age is XXXX. This means that your lung function is what would be expected for someone who is age XXXXX. This means that your lungs are ageing faster than normal. Quitting smoking cessation would stop this accelerated decline, and the lungs will start to age normally again.”*

TO ALL PATIENTS

*“This type of lung function test does not tell us anything about the risk of other serious diseases related to smoking such as lung cancer or heart disease or stroke. Smoking cessation is therefore still important for all people regardless of their age or the results of these lung tests.”*

# Supplementary file 3

# Protocol for conducting CO test and explaining significance of the result to the participant

### Procedure

1. Both patients and practitioners should use a non-alcohol sanitiser gel on their hands before the test
2. Explain background information to the patient
3. Attach the adaptor and a clean, disposable, mouthpiece (a fresh one for each patient) to the monitor
4. Turn the machine on
5. Briefly explain what you would like your patient to do
6. Ask patient to take a deep breath and begin the countdown
7. The monitor will count down 15 seconds and beep during the last 3 seconds
8. The patient needs to exhale slowly (not blow hard) into the mouthpiece aiming to empty their lungs completely
9. The parts per million (ppm) of carbon monoxide in the lungs will be displayed on the screen
10. The mouthpiece can be disposed of by the patient (they can remove the mouthpiece themselves and place it in a bin)

| **Note: Alcohol wipes should not be used on the CO monitors as the cells within them react to alcohol fumes; the same applies to alcohol hand gels. Use non-alcohol based wipes and gels to clean the monitors and your hands between patients** |
| --- |

Infection control procedures generally include:

1. Washing your hands and/or using a non-alcohol sanitising gel before and after carrying out the CO test
2. Asking the patient to place the disposable cardboard tube onto the plastic mouthpiece adaptor prior to the test
3. Asking the patient to dispose of the cardboard tube once the test has been completed
4. Wiping the CO monitor with a non-alcohol wipe between CO tests

### Suggested phrases to patient – Baseline visit

**Prior to test – background information**

*“Carbon monoxide is a gas inhaled by smokers when they smoke a cigarette and it causes heart disease. The good news for you is that shortly after stopping smoking the level of carbon monoxide in your body returns to that of a non-smoker. This machine measures the amount of carbon monoxide in your lungs in parts per million and if you have not been smoking then we would expect it to be below 10 parts per million.”*

**During the test**

*“What I am going to ask you to do in a minute is to take a big deep breath, hold your breath and then exhale into this machine. You will need to hold your breath for about 15 seconds. After you have taken your breath I will hand the machine to you, the machine will count down and I will then tell you when to exhale into it.”*

*“I’d like you to take a nice big breath.….well done……keep holding your breath, only 10 seconds left now……OK, take hold of the machine……place your lips around the tube and 3,2,1….blow now.”*

**After the test – Explaining the significance of the test results**

If the test wasn’t completed adequately (i.e. client did not hold their breath for the required time or did not place their lips around the tube properly):

*“I am afraid that we are going to need to do that again to get a proper reading. Catch your breath and then we will try it again.”*

Repeat instructions from above.

If the test was completed adequately:

*The monitor is showing a reading of XX parts per million.*

*If you were to quit smoking, the level of CO would drop. Our bodies produce small amounts of carbon monoxide and so the reading will probably not be zero; it will also fluctuate slightly depending upon what air you have been exposed to. A reading of below 10 parts per million is considered to be that of a non-smoker.*

### Suggested phrases to patient – Behavioural support visits

### (VBA+CO arm only)

**During the test**

*“We are now going to test your levels of exhaled CO. As we did before, in a minute I am going to ask you to take a big deep breath, hold your breath and then exhale into this machine. You will need to hold your breath for about 15 seconds. After you have taken your breath I will hand the machine to you, the machine will count down and I will then tell you when to exhale into it.”*

*“I’d like you to take a nice big breath.….well done……keep holding your breath, only 10 seconds left now……OK, take hold of the machine……place your lips around the tube and 3,2,1….blow now.”*

**After the test – Explaining the significance of the test results**

If the test wasn’t completed adequately (i.e. client did not hold their breath for the required time or did not place their lips around the tube properly):

*“I am afraid that we are going to need to do that again to get a proper reading. Catch your breath and then we will try it again.”*

Repeat instructions from above.

If the test was completed adequately

1. Tell the patient their reading

*“The monitor is showing a reading of XX parts per million.”*

2. Establish when the patient last had a cigarette.

**CO readings that confirm self-report**

If the CO reading is below 10 parts per million (ppm) then you can congratulate your patient on not smoking and on achieving a 'non-smokers' reading.

Contrast this reading with the CO levels measured at the pre-quit and quit date appointments and use this to further motivate the client to continue with their quit attempt.

**Discrepancies between CO readings and self-report**

If the CO monitor is reading above 10ppm, and your patient is telling you they haven't smoked, you should tell them that most smokers who report not having smoked at all in the past week have a CO reading of 10ppm or less.

There can be some exceptions however, such as in:

- Smokers who are lactose intolerant (allergic to dairy products);
- Exposure to carbon monoxide from a faulty car exhaust;
- Exposure to carbon monoxide from faulty gas boiler;
- Using a chemical paint stripper.

Lactose intolerance can produce a gas in the breath which ‘confuses’ the CO monitor. However, most people know that they are lactose intolerant as they will experience a stomach upset after consuming dairy products.

Encourage the smoker to return for the next appointment having not smoked to register a CO reading of 10ppm or less.

Typically the practitioner will say:

1. *'You have reported not smoking and I am not going to disbelieve you. So what I suggest is that you get your gas boiler/car exhaust checked out this week to make sure you are not exposed to dangerous levels of carbon monoxide. Then when you come back here next week having not smoked at all your CO reading will be 10ppm or less and you can be classed as a non-smoker.'*

This is important because you need to ensure that the smoker is not exposed to dangerous levels of carbon monoxide.

**Low CO readings in smokers**

It is possible that a smoker who doesn't smoke many cigarettes and hasn't smoked for a number of hours may have a low - less than 10ppm - CO reading.

For example, this can happen with morning appointments, when the smoker hasn't smoked that day yet. In such cases it is worth stating that CO accumulates in the body during the day, and were the test to be repeated towards the end of the day there is little doubt that it would be higher.

It is also possible that a patient who is smoking less regularly, and has not smoked for a number of hours will have a low CO reading. In this case, compare the CO reading to the reading recorded at baseline, and congratulate the smoker if it is now lower.

**Carbon monoxide poisoning**

A smoker may exhibit abnormally high expired CO levels (above 100ppm). In such cases, they should be given advice about possible acute CO poisoning, and advised to attend their local Accident and Emergency department.

**Supplementary file 4**

**Exploratory analyses (validation method for quitting smoking)**

*Methods*

The primary outcome definition was:

*“Proportion of smokers who are quit at 4 weeks (7-day point prevalence self-reported abstinence, confirmed with salivary cotinine level of: (1) <10ng/ml, or (2) <100ng/ml for those who report exposure to second hand cigarette smoke in the home on a daily basis, or (3) ≥10ng/ml in those who report using nicotine replacement therapy/e-cigarettes at any timepoint during the study, irrespective of exposure to second hand cigarette smoke)”*

In an exploratory analysis, we compared alternative definitions of the primary outcome to the main definition, these alternative definitions were determined after the trial had commenced to explore the potential impact of secondhand smoke exposure and use of NRT/e-cigarettes. In the first alternative definition, 7-day point prevalence abstinence was confirmed with an exhaled CO reading of <10ppm rather than salivary cotinine, and in the second the proportion quit (7-day point prevalence self-reported abstinence) was confirmed with salivary cotinine cut offs as stated for the primary outcome but with a time window for reported exposure to second hand smoke and use of NRT/e-cigarettes of the past 4 days. CO results were concealed from participants in the CO+VBA arm.

*Results*

Very few individuals had confirmatory testing by exhaled CO and no differences were detected. Results indicated a difference in primary outcome between cotinine validated definitions using the past four day time window (this definition resulted in less validated quitters), but the number of discordant results was very small and these results should be interpreted with caution, see table S4-1.

Table S4-1. Proportion validated quit smoking at 4, 12 and 26 weeks.

|  |  | **Quitting smoking**  using the primary definition* | |  |
| --- | --- | --- | --- | --- |
|  |  | Quit smoking | Not quit smoking | Exact McNemar’s  p-value |
| **(1) Quit smoking**  confirmed with CO monitor^ | |  |  |  |
| At 4wks |  |  |  |  |
|  | Quit smoking | 4 | 0 | 1.0000 |
|  | Not quit smoking | 0 | 1 |  |
| At 12wks |  |  |  |  |
|  | Quit smoking | 12 | 2 | 0.5000 |
|  | Not quit smoking | 0 | 1 |  |
| At 26wks |  |  |  |  |
|  | Quit smoking | 1 | 3 | 0.2500 |
|  | Not quit smoking | 0 | 0 |  |
| **(2) Quit smoking**  using an exploratory definition** | |  |  |  |
| At 4wks |  |  |  |  |
|  | Quit smoking | 17 | 0 | **0.0313** |
|  | Not quit smoking | 6 | 1457 |  |
| At 12wks |  |  |  |  |
|  | Quit smoking | 20 | 0 | 0.1252 |
|  | Not quit smoking | 4 | 1447 |  |
| At 26wks |  |  |  |  |
|  | Quit smoking | 9 | 0 | **0.0002** |
|  | Not quit smoking | 13 | 1418 |  |

* Quitting smoking using the following criteria: 7-day point prevalence self-reported abstinence, confirmed with salivary cotinine level of: (1) <10ng/ml, or (2) <100ng/ml for those who report exposure to second hand cigarette smoke in the home on a daily basis, or (3) ≥10ng/ml in those who report using NRT/e-cigarettes at any timepoint, irrespective of exposure to second hand cigarette smoke.

^ Quitting smoking confirmed with CO monitor tests using the following criteria: 7-day point prevalence self-reported abstinence with an exhaled CO reading of >10ppm.

** Quitting smoking using the following criteria:7-day point prevalence self-reported abstinence, confirmed with salivary cotinine level of: (1) <10ng/ml, or (2) <100ng/ml for those who report exposure to second hand cigarette smoke indoors in the last 4 days, or (3) ≥10ng/ml in those who report using NRT/e-cigarettes in the last 4 days, irrespective of exposure to second hand cigarette smoke.

**Supplementary file 5**

**Process evaluation – CRF measures and fidelity recordings**

*Methods*

To assess fidelity to delivery of the intervention protocols, a record was made by the GPs in the CRF for all participants when the VBA, LA and CO components were delivered. GPs were also asked to record a sample of baseline visits from each arm. Components within the lung age, CO and VBA interventions were identified from the intervention protocols and were ranked with 2, 1 and 0 points by a researcher based on the recording (2= component completely delivered, 1= component partially delivered, 0= component not delivered). The average score for each recording was calculated for the lung age, CO and VBA components, and the % of recordings with the average score of 2,1, or 0 reported.

*Results*

Delivery of VBA, LA and CO protocols as captured in the CRFs was high (98.7-100% delivered). We also analysed a total of 33 recording of baseline visits. In the VBA+LA and VBA+CO arms, over 70% of the recordings scored 2 for delivery of the LA and CO components, while up to 30% only scored 2 on the VBA components, with the majority scoring 1 and around 30% not delivering the act component for participants who did not want to quit or were unsure in the VBA+CO arm. In the VBA arm, a higher proportion of the recordings scored a 2 for advice (55%) and the act component was also generally more likely to be completely delivered in line with the protocol (table S5-1, figure S5- 1-3).

Table S5-1: Quantitative fidelity measures percentage delivered

| **Arm** | **Total (with a baseline measure)** | **All VBA components (including setting a quit date and related components)** | **All VBA components (Excluding the quit date related components)** | **All CO components** | **All LA components** |
| --- | --- | --- | --- | --- | --- |
| **VBA only** | 454 | 16 (3.5%) | 452 (99.6%) | - | - |
| **VBA +CO** | 445 | 22 (4.9%) | 444 (99.8%) | 440 (98.9%) | - |
| **VBA+LA*** | 451 | 17 (3.8%) | 445 (98.7%) | - | 451 (100%) |

* one person was not given the intervention and withdrew from the study, but gave consent for their data to be used in the analysis of the study

Figure S5- 1 – Percentage of recording scoring 0, 1 or 2 for intervention fidelity for each intervention component in the VBA arm (VBA)

Figure S5- 2: Percentage of recording scoring 0, 1 or 2 for intervention fidelity for each intervention component in the lung age arm (LA+VBA)

Figure S5-3 : Percentage of recording scoring 0, 1 or 2 for intervention fidelity for each intervention component in the CO arm (CO- VBA)
